# Supplementary material for: SyNDI: synchronous network data integration framework
Source: BMC Bioinformatics. 2018 Nov 6;19:403. doi: 10.1186/s12859-018-2426-5 (PMC6219086; doi:10.1186/s12859-018-2426-5)
Supplement: Supplementary file 5 — Files of the example presented in Additional file 4. This additional file is a zip package that contains all files used in Additional file 4. Detailed descriptions of each of these files are presented in a README file included in this zip package. (GZ 30411 kb) [file 12859_2018_2426_MOESM5_ESM.gz › SyNDITools/Meme2Fimo/meme_4.9.0_4.tar.gz!/meme_4.9.0/website/html/memechip-intro.html]

MEME - Overview


|  |  |
| --- | --- |
|  | MEME Suite The MEME Suite web application requires the use of JavaScript   Javascript doesn't seem to be available on your browser.  Overview  MEME-ChIP is an ensemble of tools for discovering **motifs** in a large set of related DNA sequences.  MEME-ChIP takes as input FASTA-formatted DNA sequences, of arbitrary size and number of sequences. It assumes that a technology has been used that results in sequences sufficiently close to centered on a likely TF binding location that it makes sense to focus on the central 100 bp portion of each sequence. ChIP-seq is an example of such a technology. The tools output the number of motifs as requested on the input form using MEME, and as many motifs as pass the threshold tests of the other tools.  Your MEME-ChIP results consist of multiple outputs in the formats described in a top-level summary file.  MEME-ChIP uses a combination of motif discovery using MEME (good for wide motifs) and DREME (good for shorter motifs) and comparison of both found motifs and the sequence data against databases of known motifs. |
